# Supplementary material for: Interleukin‐12/23 deficiency differentially affects pathology in male and female Alzheimer's disease‐like mice
Source: EMBO Rep. 2020 Jan 30;21(3):e48530. doi: 10.15252/embr.201948530 (PMC7054677; doi:10.15252/embr.201948530)
Supplement: Supplementary file 1 — Appendix [file EMBR-21-e48530-s001.pdf]

Appendix - Table of contents

Appendix Figure S1. 2

Appendix Figure S2. 3

Appendix Figure S3. 4

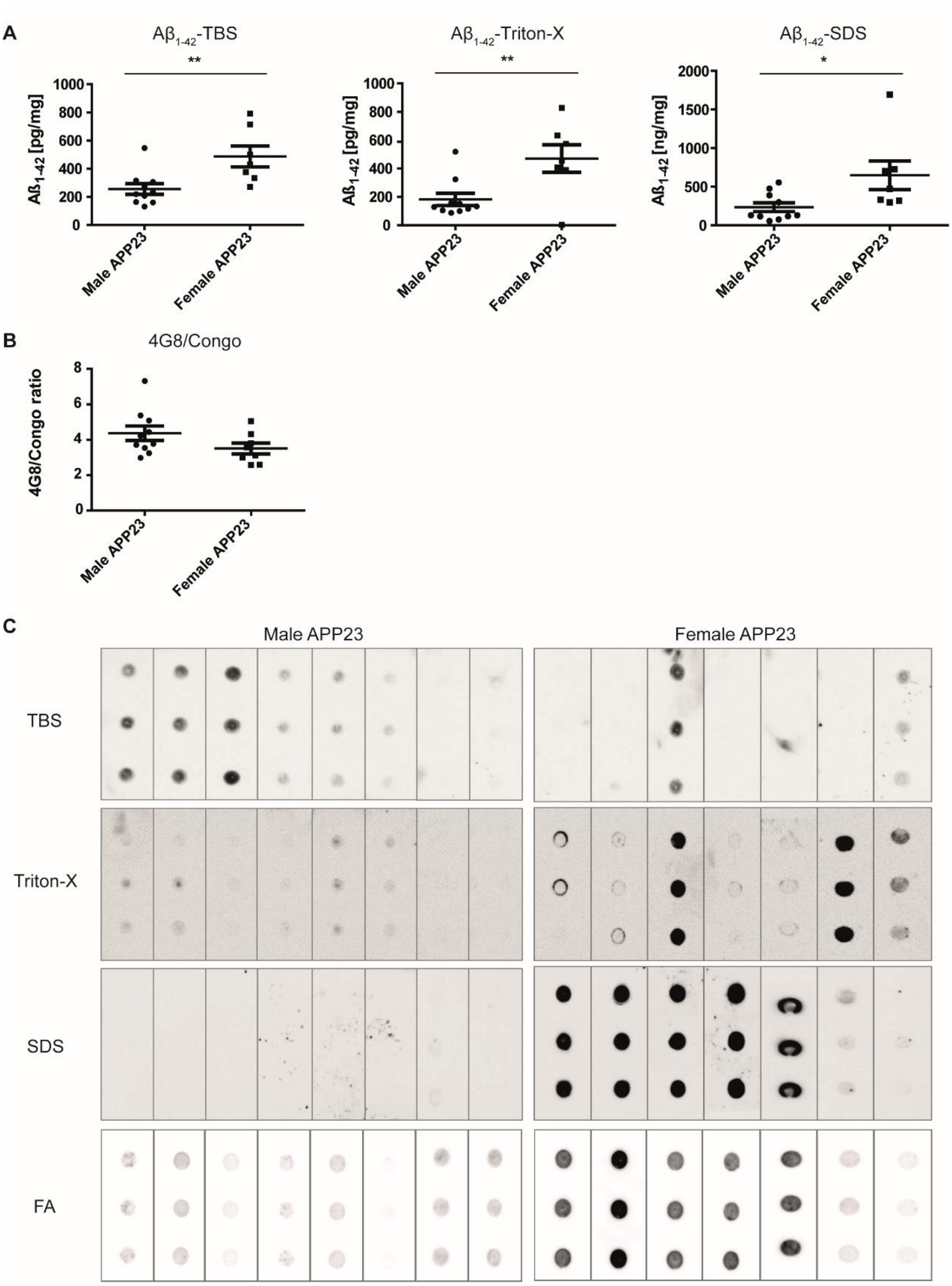

Appendix Figure S1. Levels of various Aβ species in male and female APP23 mice. (A) Mesoscale analysis of Aβ_{1-42} protein in the TBS (p=0.0082), Triton-X (p=0.0085) and SDS (p=0.0267) fractions of brain homogenates from male (n=10) and female (n=7) APP23 mice. Total protein concentration of each sample was used as an internal reference. Mean ± s.e.m., statistical analysis: two-tailed unpaired t-test. (B) Ratio of the percentage of the cortical area covered by 4G8- and Congo Red- positive amyloid plaques in male (n=10) and female (n=8) APP23 mice. Mean ± s.e.m., statistical analysis: two-tailed unpaired t-test, p=0.1235. (C) 6E10-stained dot blots of filter membranes analyzing TBS, Triton-X, SDS and formic acid (FA)-soluble protein fractions in male (left, n=8) and female (right, n=7) APP23 mice. Each sample was analysed in triplicate, each box representing one sample.

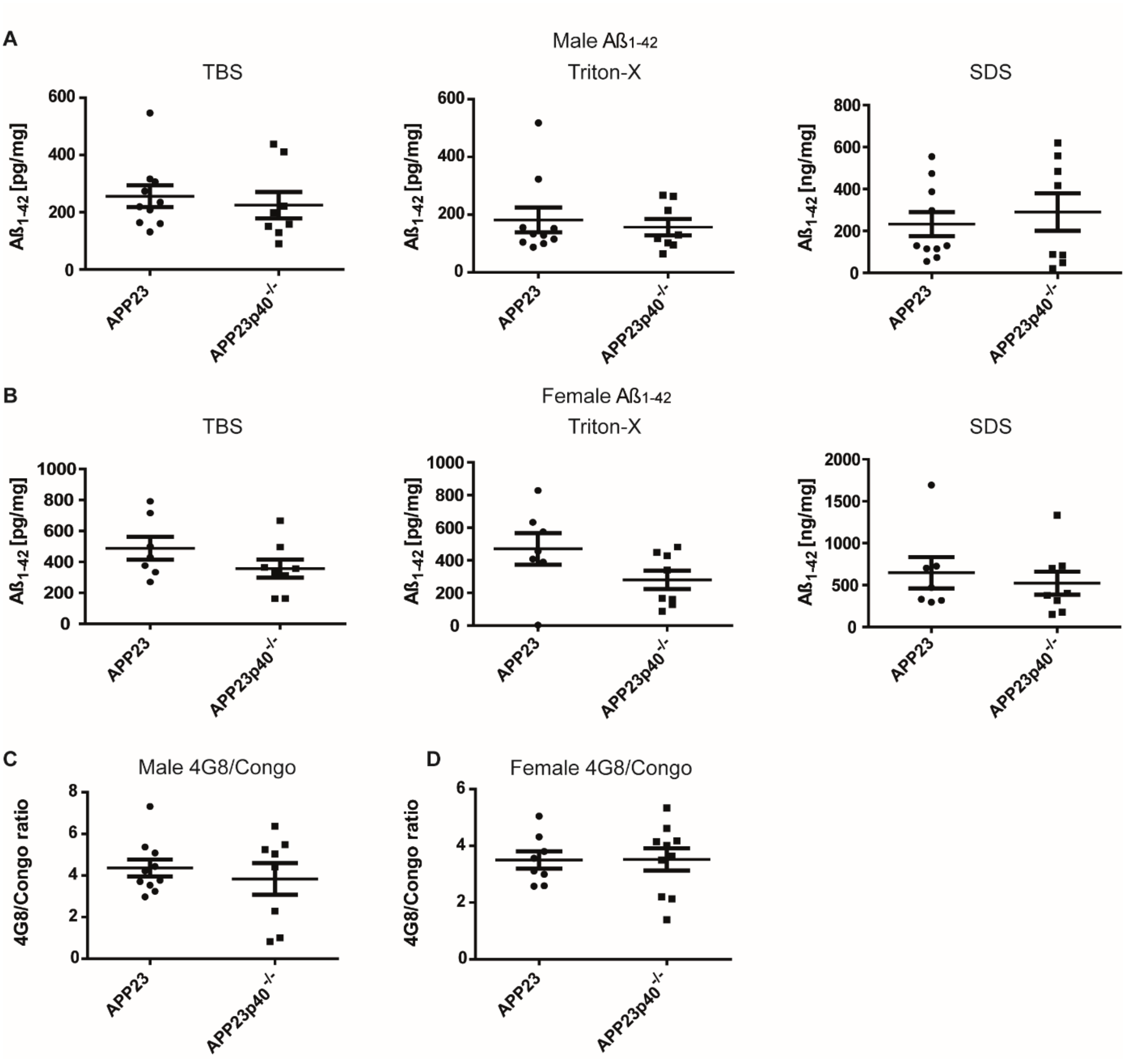

Appendix Figure S2. Effect of IL12p40 deficiency on various Aβ species. (A-B) Mesoscale analysis of Aβ_{1-42} protein in the TBS (male p=0.6029, female p=0.1822), Triton-X (male p=0.6506, female p=0.1049) and SDS (male p=0.5841, female p=0.5945) fractions of brain homogenates from (A) male APP23 (n=10) and APP23p40$^{-/-}$ (n=8) mice and from (B) female APP23 (n=7) and APP23p40$^{-/-}$ (n=8) mice. Total protein concentration of each sample was used as an internal reference. Mean ± s.e.m., statistical analysis: two-tailed unpaired t-test. (C-D) Ratio of the percentage of the cortical area covered by 4G8- and Congo Red- positive amyloid plaques in (C) male APP23 (n=10) and APP23p40$^{-/-}$ (n=8) mice (p=0.5236) and in (D) female APP23 (n=8) and APP23p40$^{-/-}$ (n=10) mice (p=0.9749). Mean ± s.e.m., statistical analysis: two-tailed unpaired t-test.

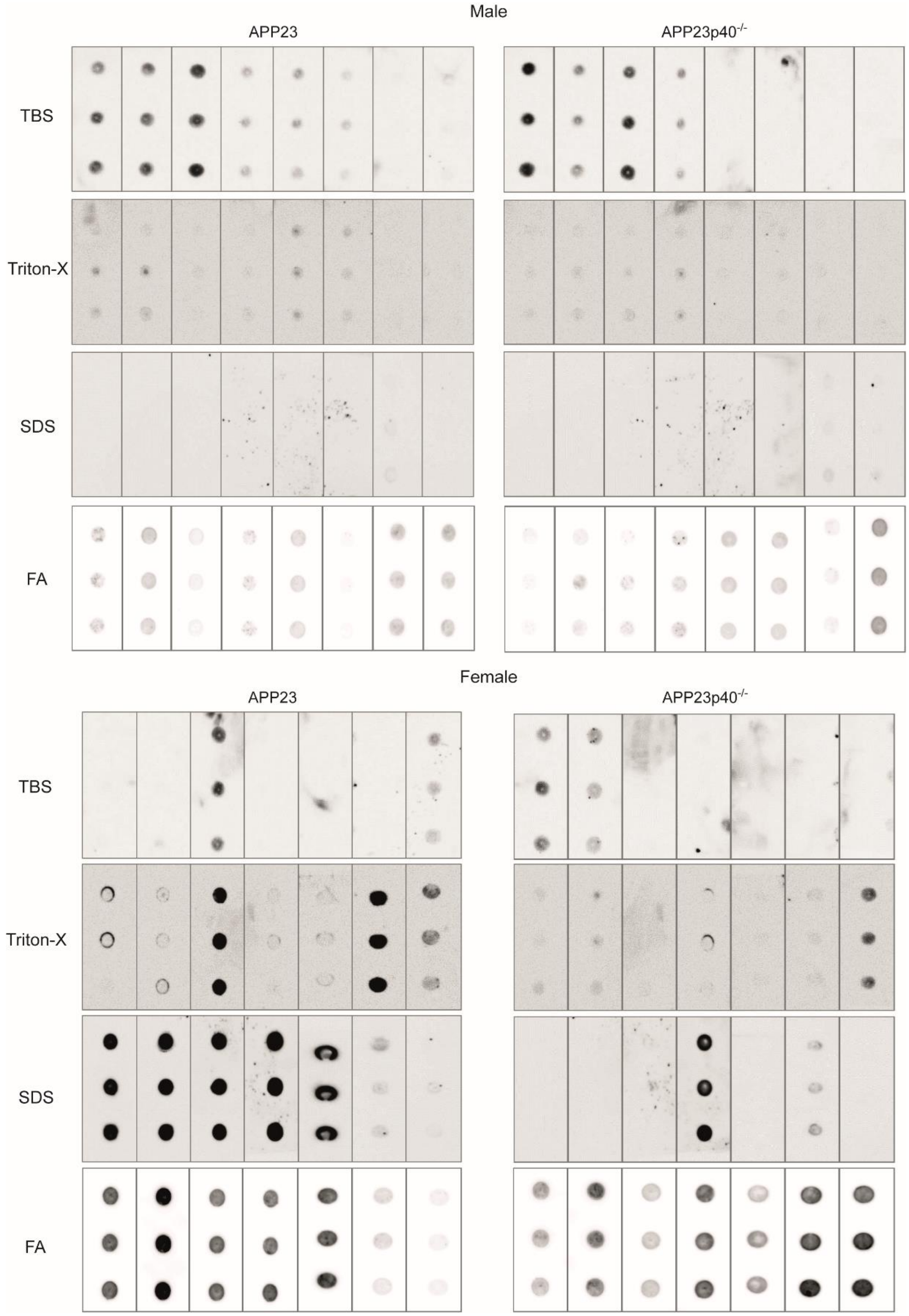

Appendix Figure S3. Filter assay of brain homogenates from male and female APP23 and APP23p40$^{-/-}$ mice. 6E10-stained dot blots of filter membranes analysing TBS, Triton-X, SDS and formic acid (FA)-soluble protein fractions in male APP23 (n=8) and APP23p40$^{-/-}$ (n=8) mice (top) and female APP23 (n=7) and APP23p40$^{-/-}$ (n=7) mice (bottom). Each sample was analysed in triplicate, each box representing one sample.
