# Supplementary material for: Interleukin‐12/23 deficiency differentially affects pathology in male and female Alzheimer's disease‐like mice
Source: EMBO Rep. 2020 Jan 30;21(3):e48530. doi: 10.15252/embr.201948530 (PMC7054677; doi:10.15252/embr.201948530)
Supplement: Supplementary file 2 — Expanded View Figures PDF [file EMBR-21-e48530-s002.pdf]

Expanded View Figures

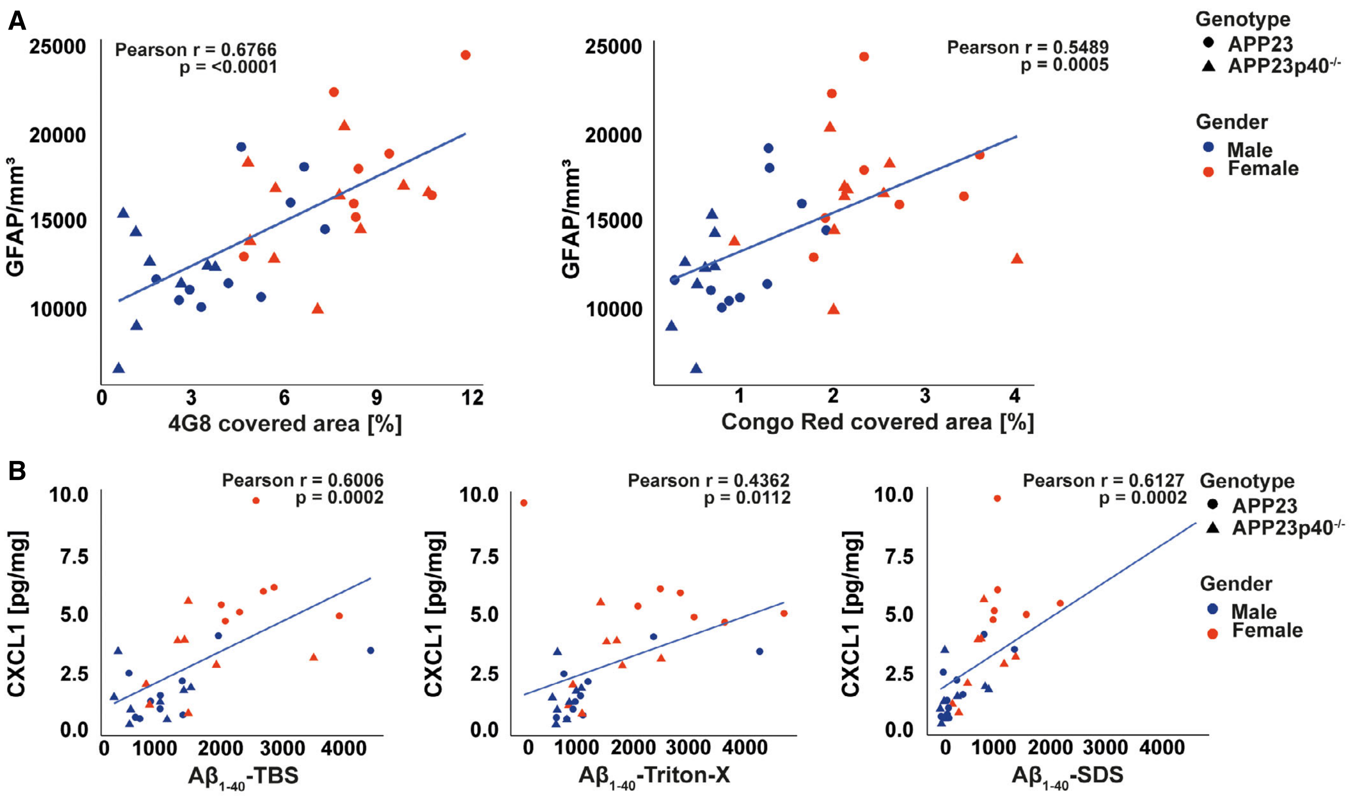

Figure EV1. Aβ pathology correlates with astrocyte number and with CXCL1 levels.

A Correlation analysis between the number of cortical astrocytes and the cortical area covered by 4G8 (left, P = < 0.0001) and Congo Red (right, P = 0.0005) in male and female APP23 (n = 8; n = 7, respectively) and APP23p40$^{-/-}$ mice (n = 8 for both genders). Statistical analysis: correlation analysis.

B Correlation analysis between the CXCL1 levels in the brain and Aβ_{1-40} levels in TBS (left, P = 0.0002), Triton-X (middle, P = 0.0112) and SDS (right, P = 0.0002) protein fractions of male and female APP23 (n = 8; n = 7, respectively) and APP23p40$^{-/-}$ mice (n = 8 for both genders). Statistical analysis: correlation analysis.

© 2020 The Authors

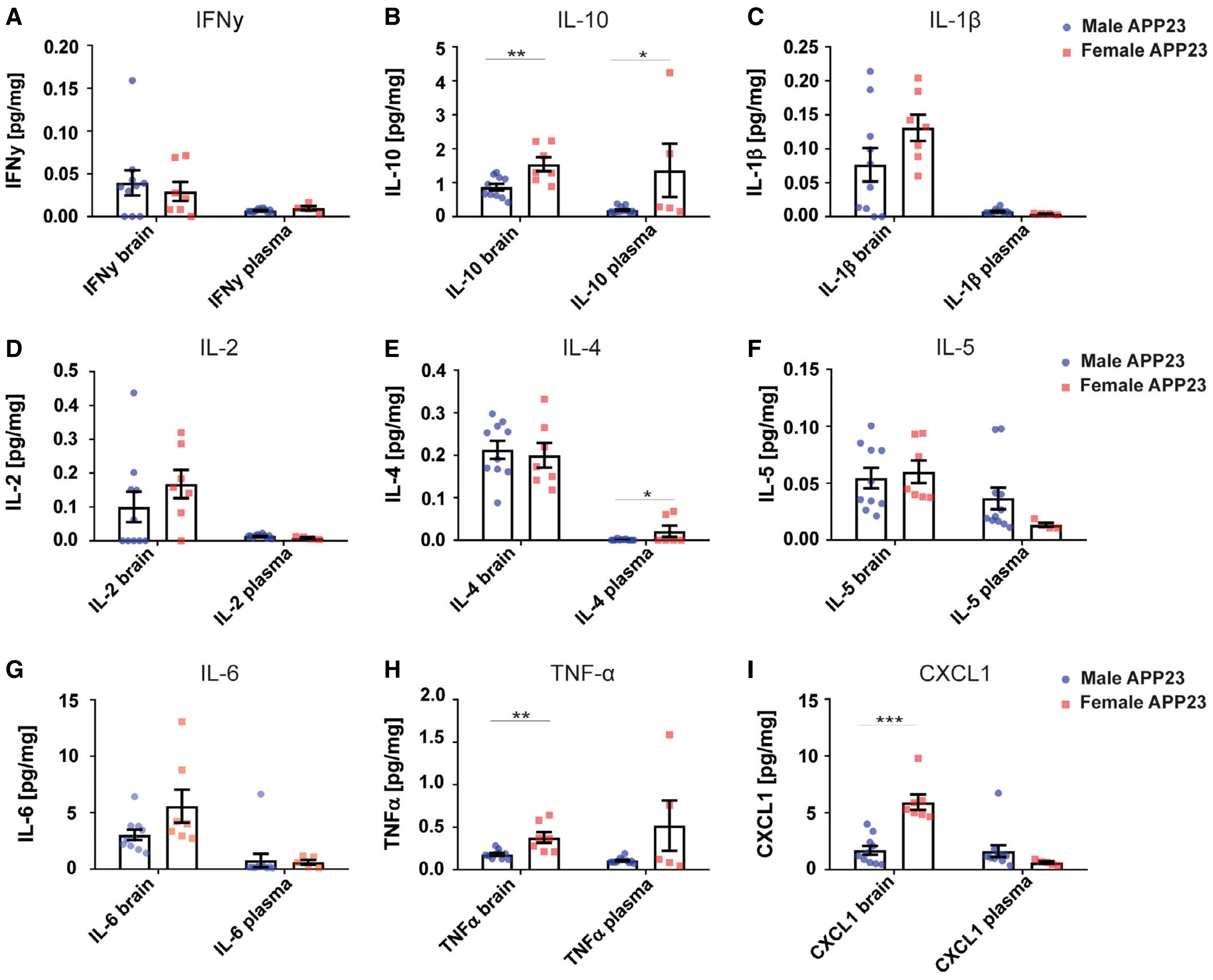

Figure EV2. Pro- and anti-inflammatory cytokines are differentially modulated between male and female APP23 mice.

A–I V-PLEX analysis for (A) IFNγ (brain P = 0.6166, plasma P = 0.1683), (B) IL-10 (brain **P = 0.0055, plasma *P = 0.0294), (C) IL-1β (brain P = 0.1267, plasma P = 0.0814), (D) IL-2 (brain P = 0.3095, plasma P = 0.0638), (E) IL-4 (brain P = 0.7099, plasma *P = 0.0220), (F) IL-5 (brain P = 0.6889, plasma P = 0.1748), (G) IL-6 (brain P = 0.0741, plasma P = 0.8609), (H) TNF-α (brain **P = 0.0031, plasma P = 0.0504) and (I) CXCL1 (brain ***P = < 0.0001, plasma P = 0.2221) protein levels in the TBS fraction of brain homogenates and plasma samples from male (brain n = 10, plasma n = 11–12) and female (brain n = 7, plasma n = 4–5) APP23 mice. Total protein concentration of each sample was used as an internal reference and some plasma values removed based on the Grubbs's outlier test. Mean ± SEM, statistical analysis: two-tailed unpaired t-test between brain and serum, respectively.

 © 2020 The Authors

Figure EV3. In male APP23p40$^{-/-}$ mice, Aβ processing is unchanged compared to APP23 mice, while serum IFNγ levels are reduced.

A Western blot analysis of APP levels in SDS-soluble protein homogenates in male APP23 (n = 7) and APP23p40$^{-/-}$ (n = 7) mice. APP expression levels were normalised to β-Actin. Mean ± SEM, statistical analysis: two-tailed unpaired t-test, P = 0.6702.

B Western blot analysis of insulin-degrading enzyme (IDE) (P = 0.9237), Neprilysin (Nep) (P = 0.7154) and BACE1 (P = 0.6421) levels in Triton-X-soluble protein homogenates in male APP23 (n = 4) and APP23p40$^{-/-}$ (n = 4) mice. Protein expression levels were normalised to GAPDH. Samples not showing a positive signal for GAPDH due to low protein content were excluded from analysis. Mean ± SEM, statistical analysis: two-tailed unpaired t-test.

C–K V-PLEX analysis for (C) IFNγ brain (P = 0.7587, plasma ***P = < 0.0001), (D) IL-10 (brain P = 0.0882, plasma P = 0.3591), (E) IL-1β (brain P = 0.9520, plasma P = 0.6272), (F) IL-2 (brain P = 0.6889, plasma P = 0.3795), (G) IL-4 (brain P = 0.9515, plasma values undetected in APP23p40$^{-/-}$ group, P-value does not apply), (H) IL-5 (brain P = 0.9591, plasma P = 0.0958), (I) IL-6 (brain P = 0.6330, plasma P = 0.4225), (J) TNF-α (brain P = 0.1194, plasma P = 0.2246) and (K) CXCL1 (brain P = 0.5305, plasma P = 0.8743) protein levels in the TBS fraction of brain homogenates and plasma samples from male APP23 (brain n = 10, plasma n = 11–12) and APP23p40$^{-/-}$ (brain n = 8, plasma n = 7–8) mice. Total protein concentration of each sample was used as an internal reference and some plasma values removed based on the Grubbs's outlier test. Mean ± SEM, statistical analysis: two-tailed unpaired t-tests between brain and serum respectively.

Source data are available online for this figure.

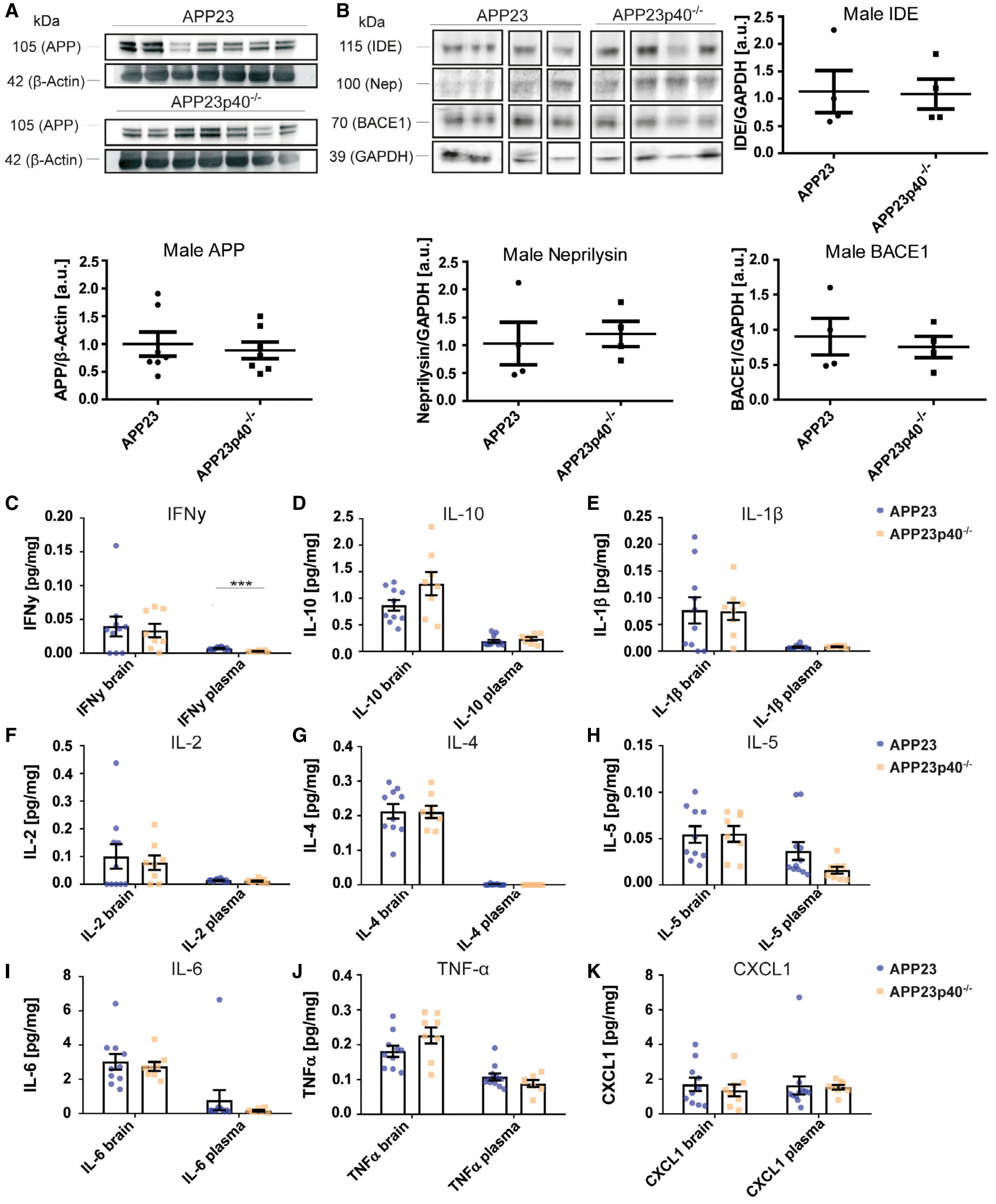

Figure EV3.

© 2020 The Authors

Figure EV4. In female APP23p40$^{-/-}$ mice, Aβ processing is unchanged compared to APP23 mice, while serum and brain cytokine levels are modified.

A Western blot analysis of APP levels in SDS-soluble protein homogenates in female APP23 (n = 7) and APP23p40$^{-/-}$ (n = 7) mice. APP expression levels were normalised to β-actin. Mean ± SEM, statistical analysis: two-tailed unpaired t-test, P = 0.9375.

B Western blot analysis of insulin-degrading enzyme (IDE) (P = 0.0543), Neprilysin (Nep) (P = 0.1601) and BACE1 (P = 0.9704) levels in Triton-X-soluble protein homogenates in female APP23 (n = 4) and APP23p40$^{-/-}$ (n = 5) mice. Protein expression levels were normalised to GAPDH. Samples not showing a positive signal for GAPDH due to low protein content were excluded from analysis. Mean ± SEM, statistical analysis: two-tailed unpaired t-test.

C–K V-PLEX analysis for (C) IFNγ (brain P = 0.4896, plasma P = 0.0922), (D) IL-10 (brain P = 0.4754, plasma P = 0.5060), (E) IL-1β (brain P = 0.3014, plasma **P = 0.0099), (F) IL-2 (brain P = 0.2530, plasma P = 0.0766), (G) IL-4 (brain P = 0.2884, plasma P = 0.0818), (H) IL-5 (brain P = 0.4128, plasma *P = 0.0421), (I) IL-6 (brain P = 0.2593, plasma *P = 0.0181), (J) TNF-α (brain P = 0.8305, plasma P = 0.1776) and (K) CXCL1 (brain **P = 0.0033, plasma *P = 0.0339) protein levels in the TBS fraction of brain homogenates and plasma samples from female APP23 (brain n = 7, plasma n = 4–5) and APP23p40$^{-/-}$ (brain n = 8, plasma n = 7) mice. Total protein concentration of each sample was used as an internal reference and some plasma values removed based on the Grubbs's outlier test. Mean ± SEM, statistical analysis: two-tailed unpaired t-test between brain and serum respectively.

Source data are available online for this figure.

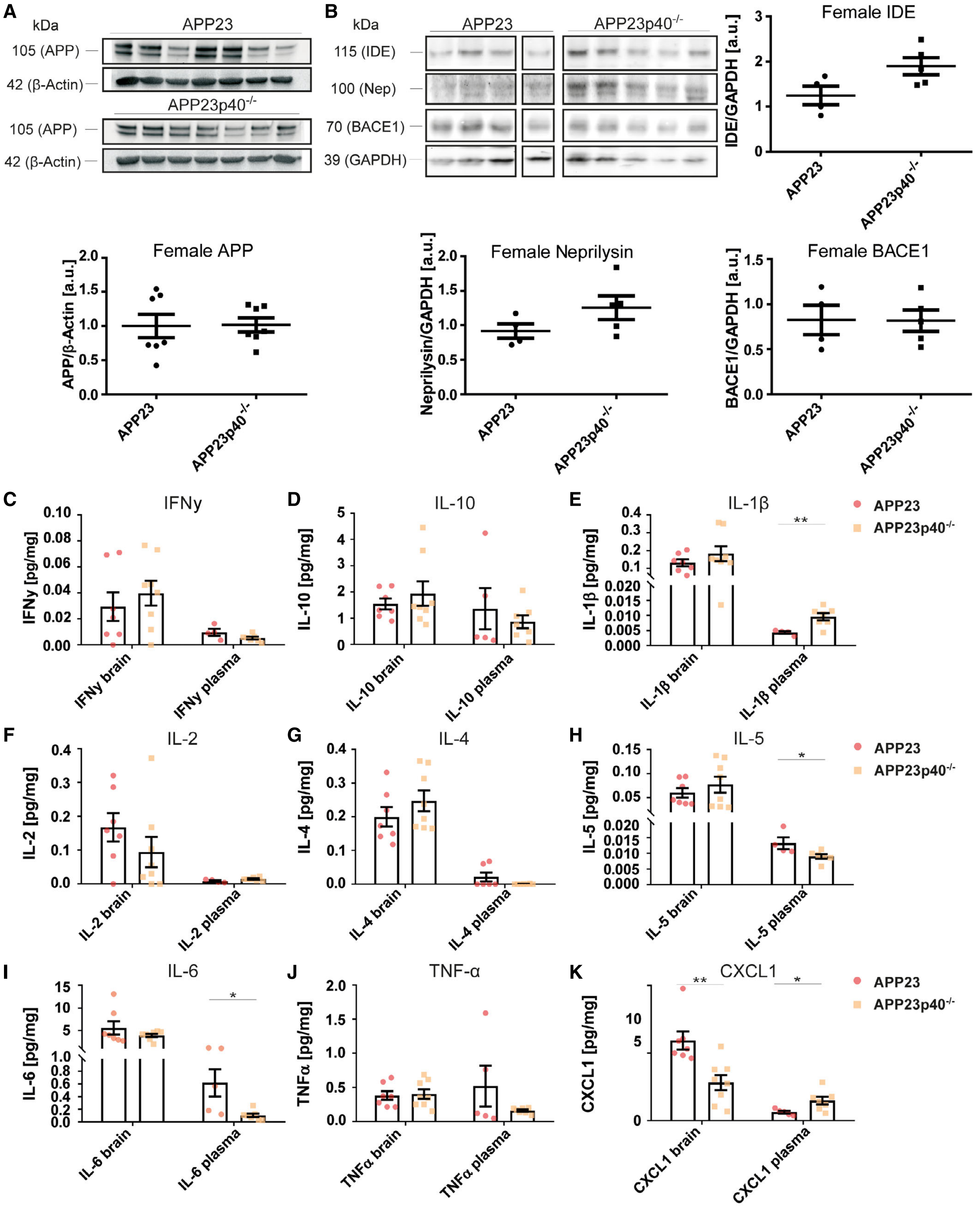

Figure EV4.

 © 2020 The Authors
