# Supplementary figures and images for: Interleukin‐12/23 deficiency differentially affects pathology in male and female Alzheimer's disease‐like mice
Source: EMBO Rep. 2020 Jan 30;21(3):e48530. doi: 10.15252/embr.201948530 (PMC7054677; doi:10.15252/embr.201948530)

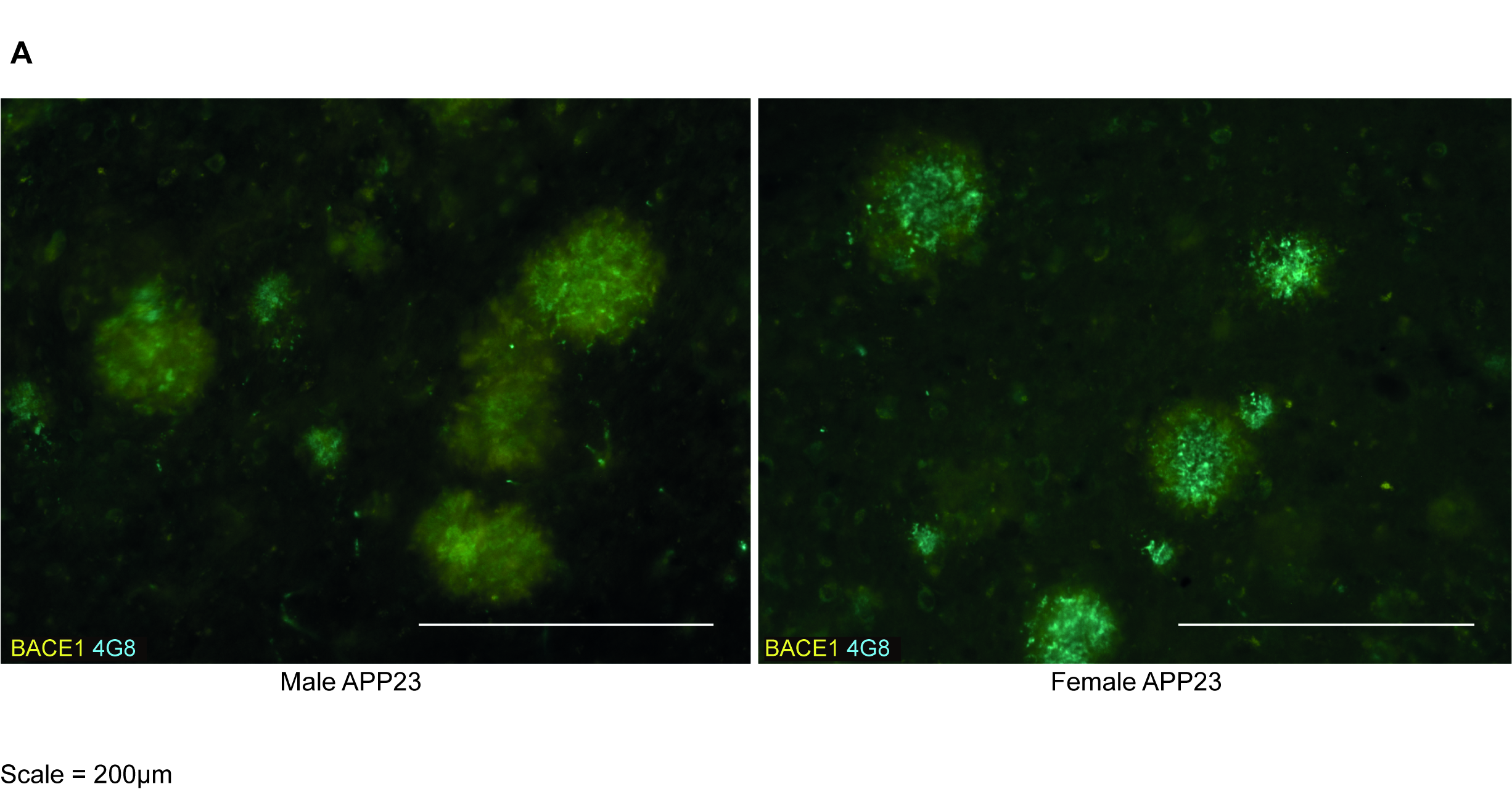

Supplement: Supplementary file 3 — Source Data for Figure 2 [file EMBR-21-e48530-s003.zip › embr201948530-sup-0003-SDataFig2A.tif]

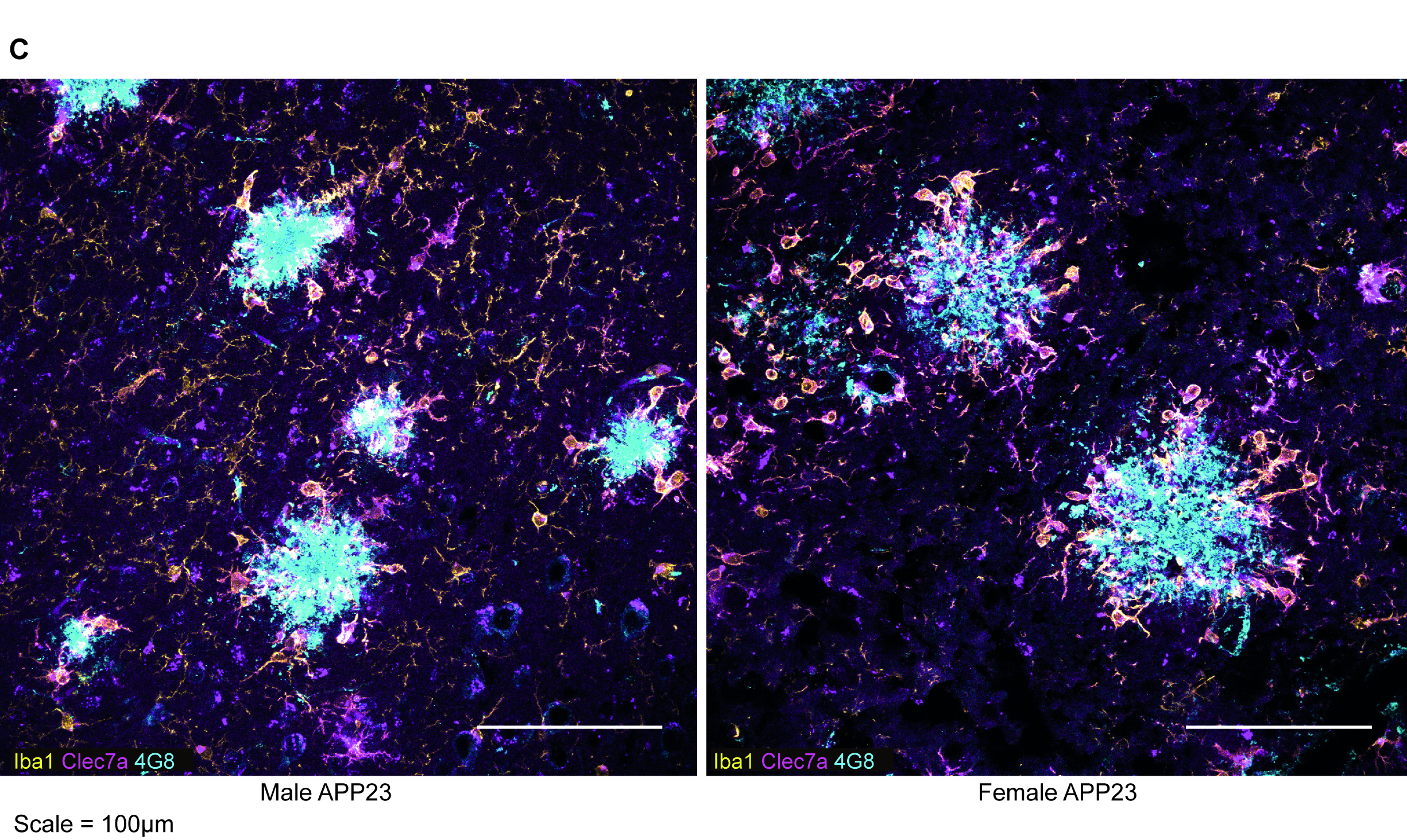

Supplement: Supplementary file 3 — Source Data for Figure 2 [file EMBR-21-e48530-s003.zip › embr201948530-sup-0004-SDataFig2C.tif]

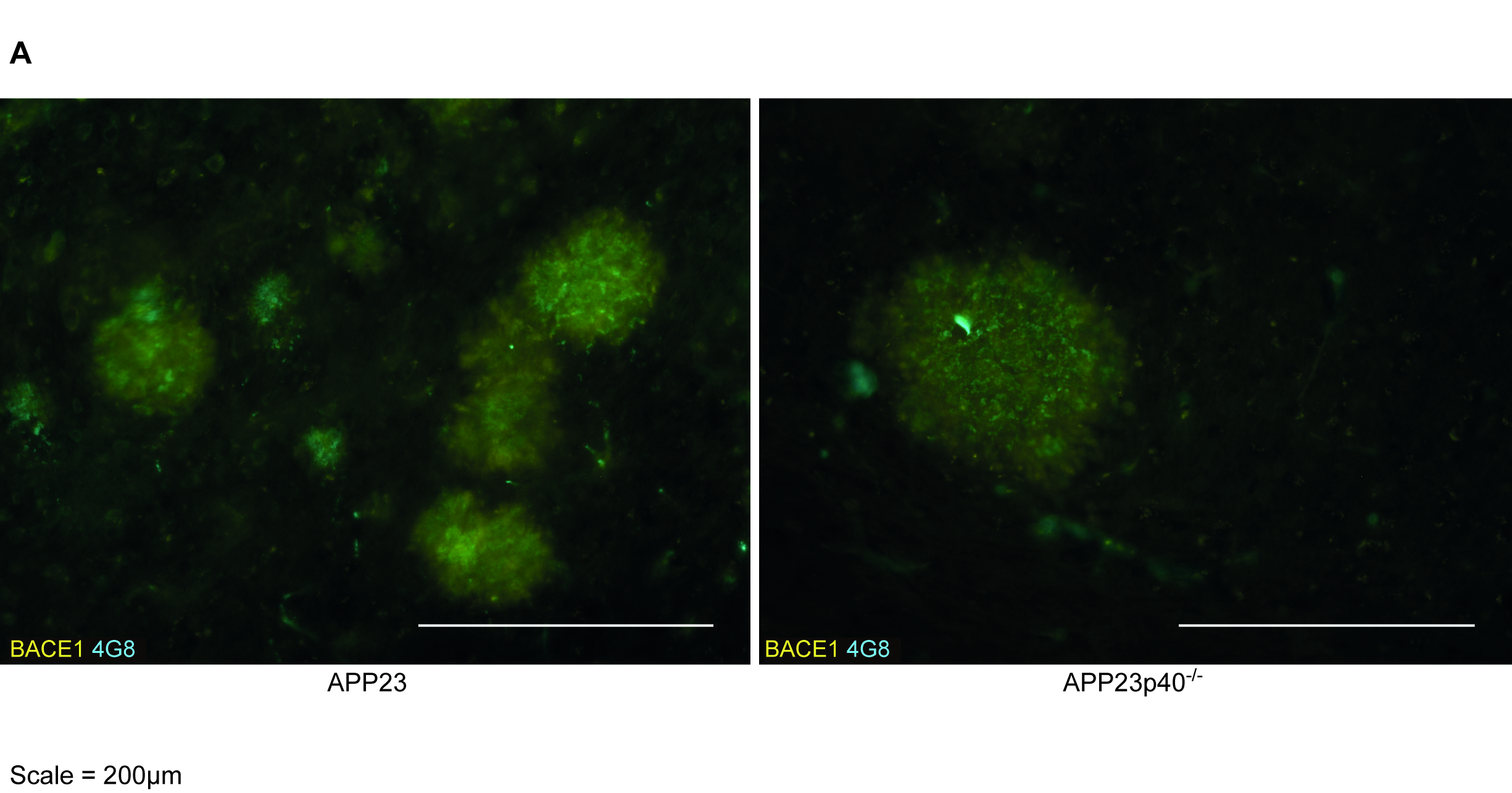

Supplement: Supplementary file 4 — Source Data for Figure 5 [file EMBR-21-e48530-s004.zip › embr201948530-sup-0005-SDataFig5A.tif]

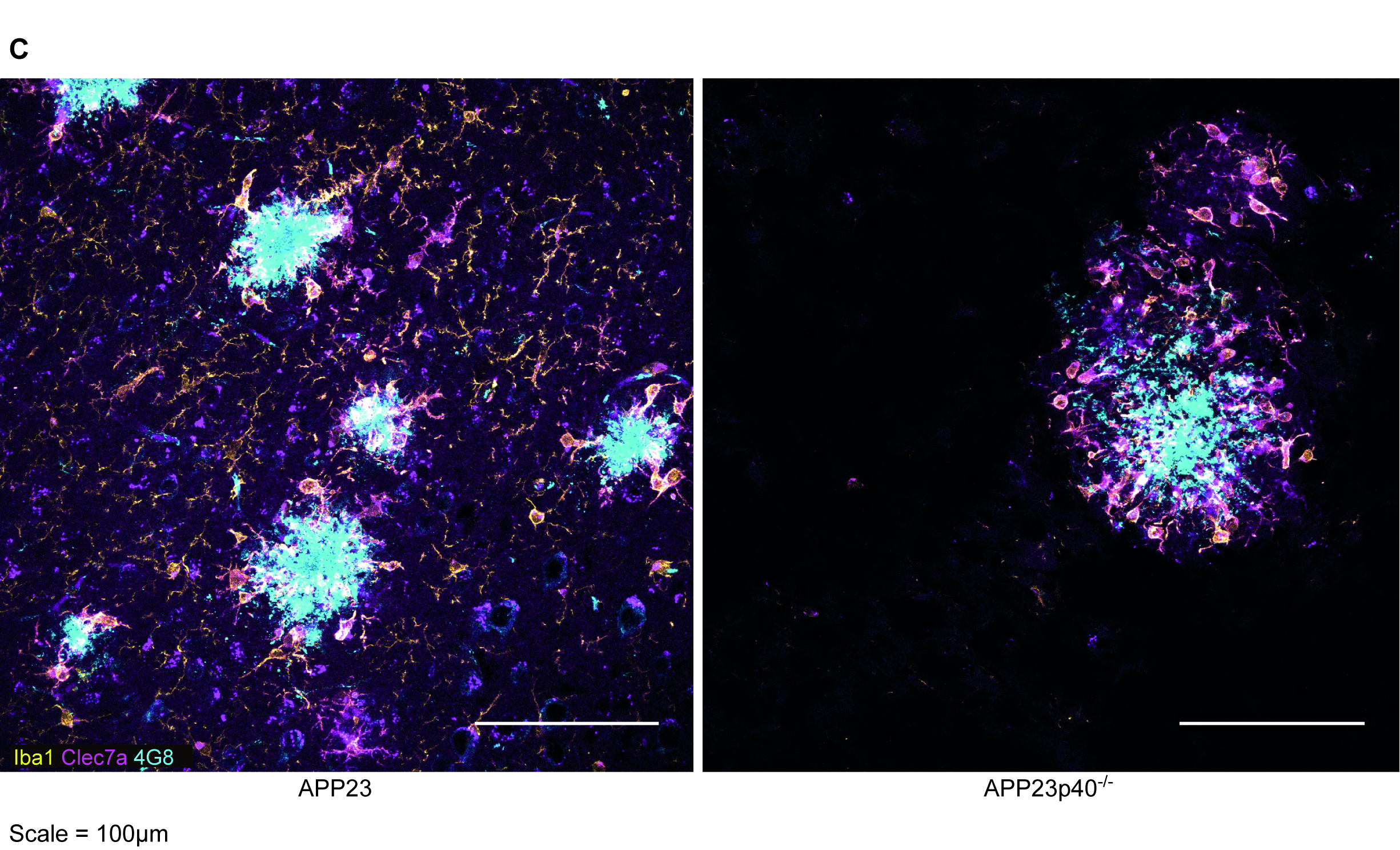

Supplement: Supplementary file 4 — Source Data for Figure 5 [file EMBR-21-e48530-s004.zip › embr201948530-sup-0006-SDataFig5C.tif]

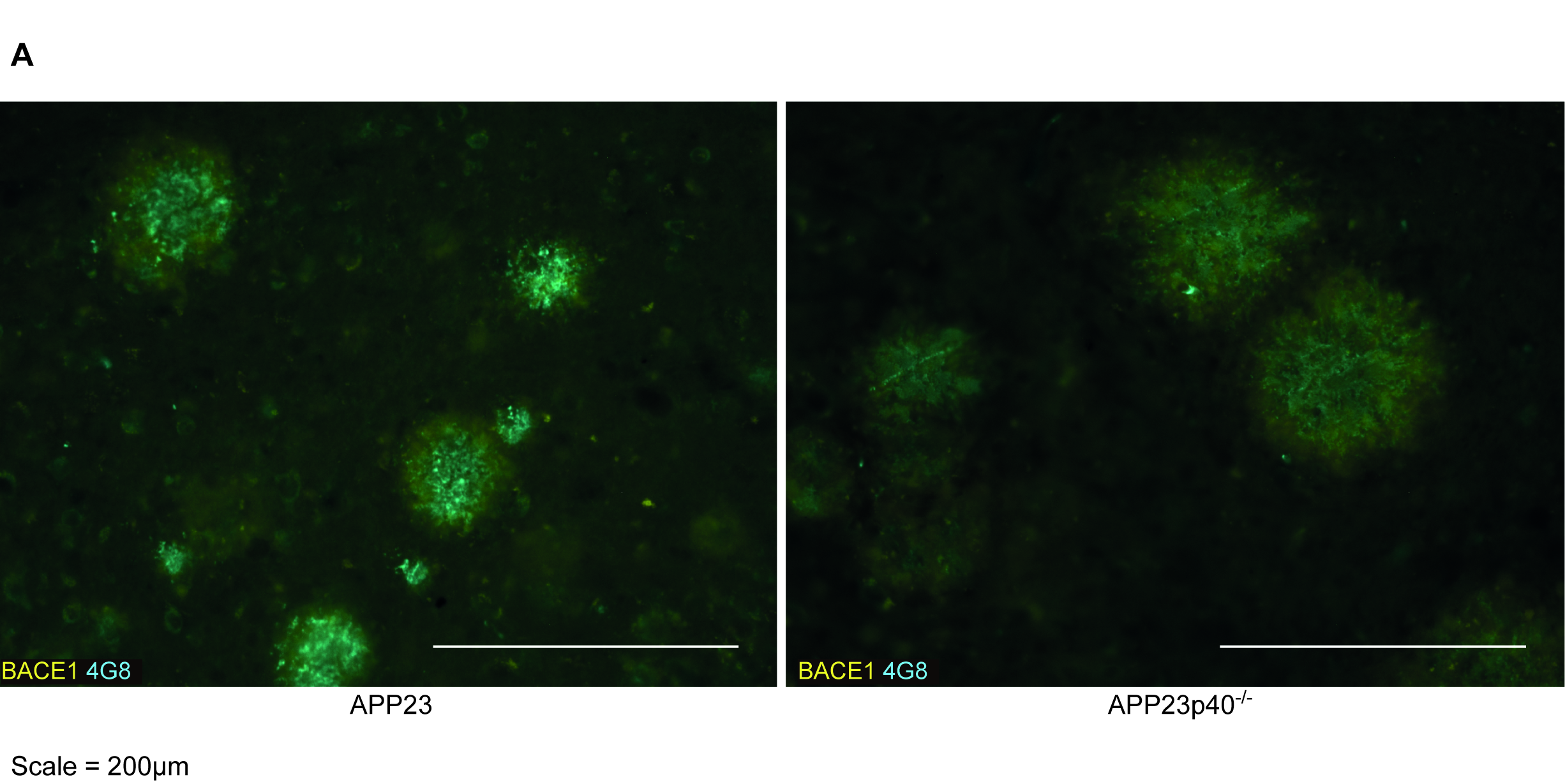

Supplement: Supplementary file 5 — Source Data for Figure 7 [file EMBR-21-e48530-s005.zip › embr201948530-sup-0007-SDataFig7A.tif]

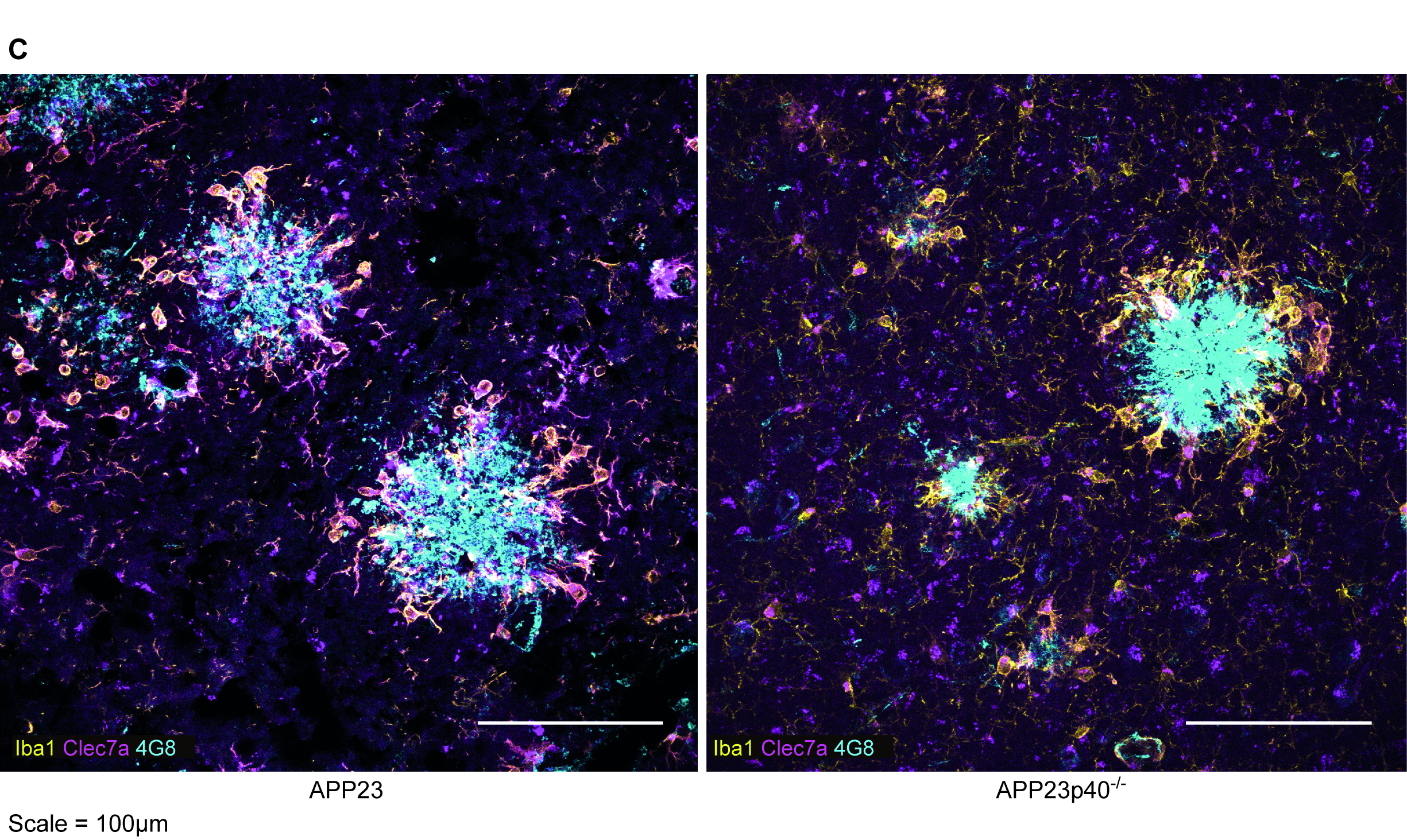

Supplement: Supplementary file 5 — Source Data for Figure 7 [file EMBR-21-e48530-s005.zip › embr201948530-sup-0008-SDataFig7C.tif]

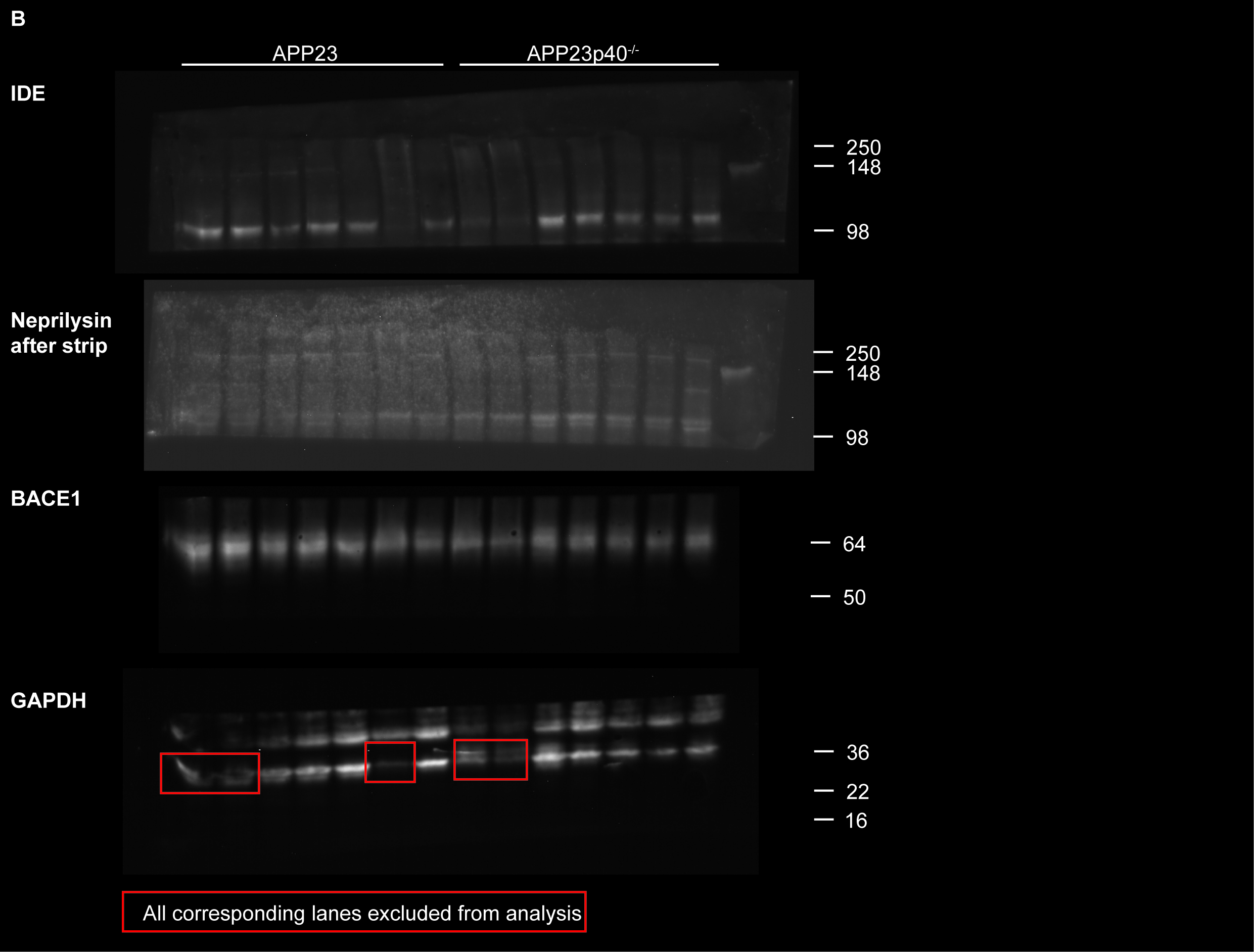

Supplement: Supplementary file 6 — Source Data for Expanded View [file EMBR-21-e48530-s006.zip › EMBOR-2019-48530V1_SourceDataForFigureEV4B.tif]

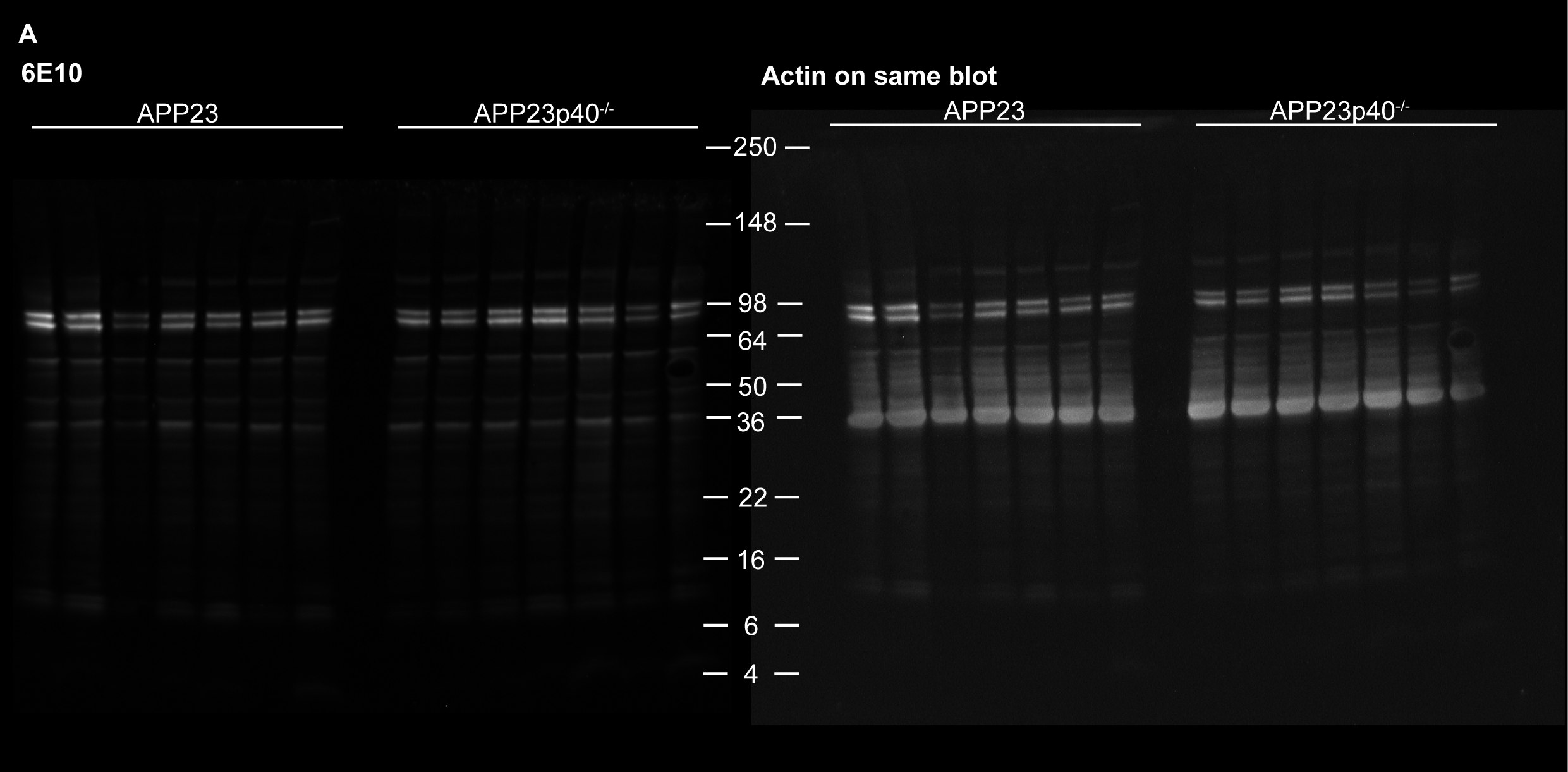

Supplement: Supplementary file 6 — Source Data for Expanded View [file EMBR-21-e48530-s006.zip › EMBOR-2019-48530V1_SourceDataForFigureEV3A.tif]

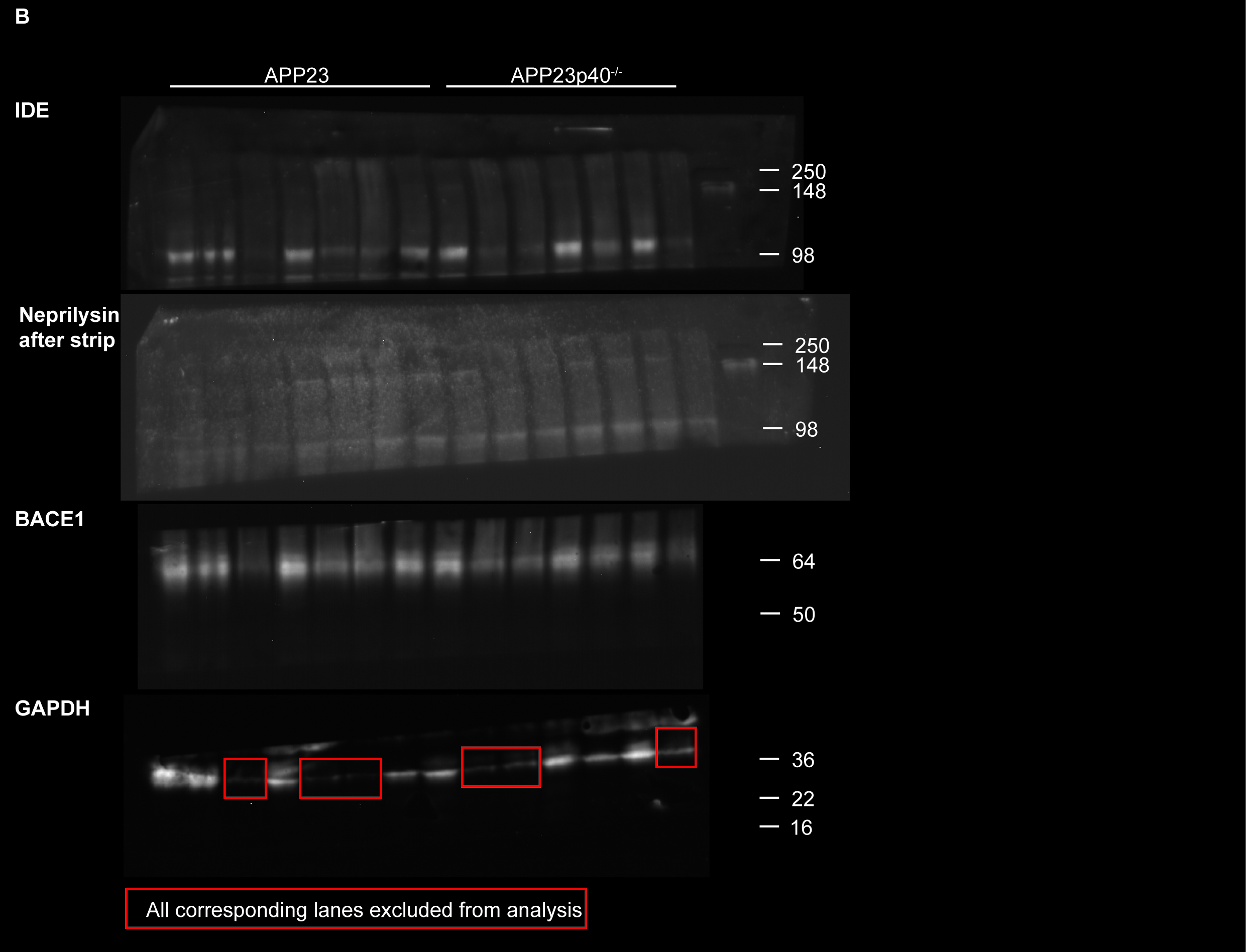

Supplement: Supplementary file 6 — Source Data for Expanded View [file EMBR-21-e48530-s006.zip › EMBOR-2019-48530V1_SourceDataForFigureEV3B.tif]

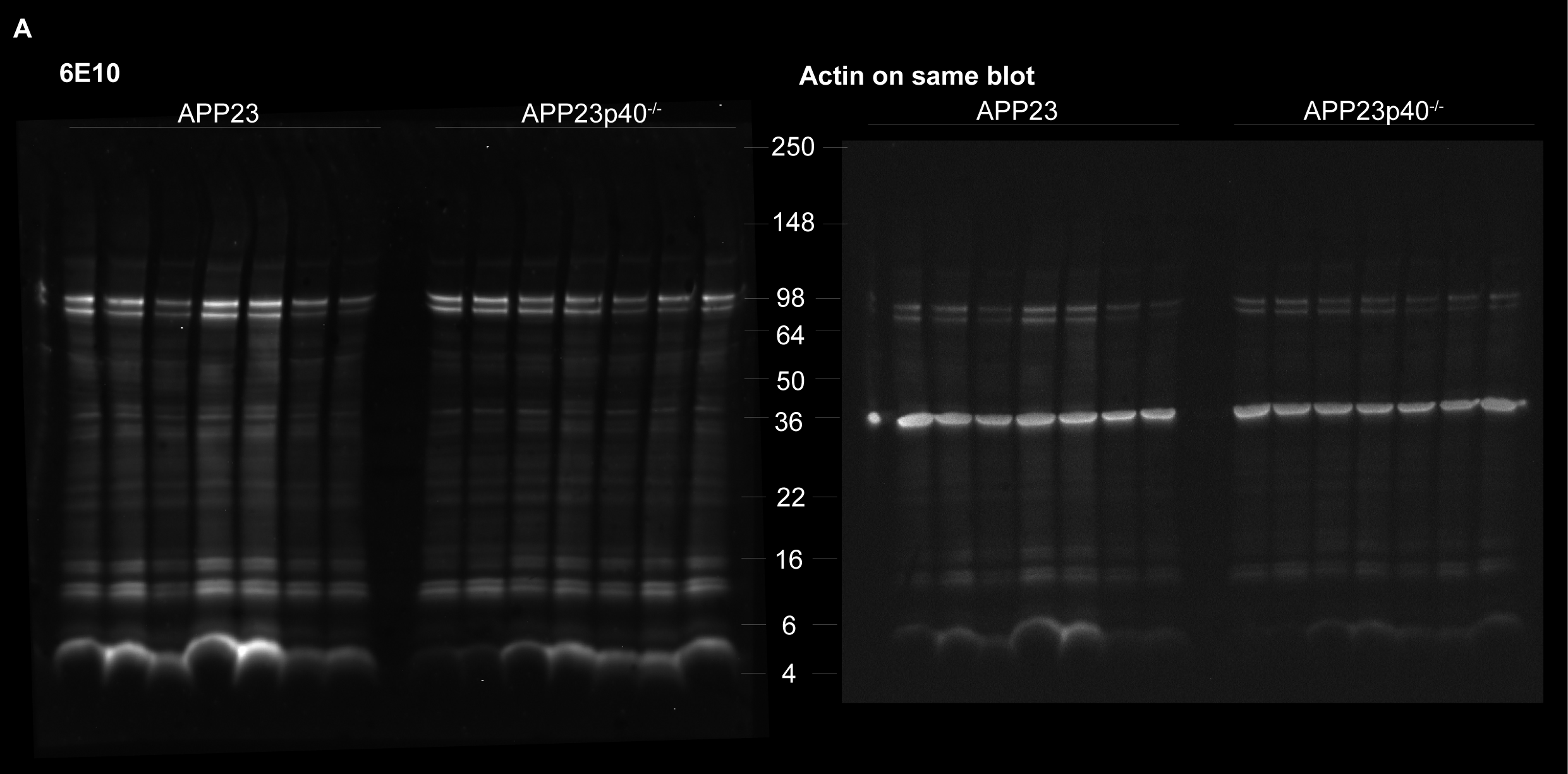

Supplement: Supplementary file 6 — Source Data for Expanded View [file EMBR-21-e48530-s006.zip › EMBOR-2019-48530V1_SourceDataForFigureEV4A.tif]
